# 

**Published:** 1893-10-07

**Authors:** 


### The Hospital

An Institutional Journal of

### The Medical Sciences and Hospital Administration.

EDITED BY

HENRY C. BURDETT,

AUTHOR OF "HOSPITALS AND ASYLUMS OF THE WORLD: THEIR ORIGIN, HISTORY, CONSTRUCTION, ADMINISTRATION, MANAGEMENT AND LEGISLATION"; "HOSPITALS AND THE STATE"; "PAY HOSPITALS OF THE WORLD"; "COTTAGE HOSPITALS, GENERAL, FEVER, AND CONVALESCENT, WITH FIFTY BEDS AND UNDER": "BURDETT'S HOSPITAL ANNUAL AND YEARBOOK OF PHILANTHROPY"; AND "THE RELATIVE MORTALITY OF LARGE AND SMALL HOSPITALS."

ACTING EDITORS:

GEORGE W. POTTER, M.D.Edin.,

AUTHOR OF "MINISTERING WOMEN"; AND NUMEROUS PAPERS ON SCIENTIFIC AND MEDICAL SUBJECTS;

AND

P. WATSON WILLIAMS, M.D.Lond.,

PHYSICIAN TO THE OUT-PATIENTS AND FOR DISEASES OF THE THROAT, BRISTOL ROYAL INFIRMARY, ETC.

INDEX TO VOL. XV.

**(30th SEPTEMBER, 1893, to 31st MARCH, 1894.)**

LONDON:
THE SCIENTIFIC PRESS (LIMITED), 428, STRAND, W.C.
1894.

SURGEON GENL'S OFFICE
150279
LIBRARY.
J76742 War, Lot 6, 22
430
Red

W1
HO69X

### INDEX TO VOL. XV., 1893-1894.

A B C Diet, 103
Aberdeen Institutions (W.), 445
Accounts, Uniform System of, for Hospitals (W.), 126
Achievements Under Suffering, Great, 478
Acidity of the Gastric Juice, On the Diagnostic Value of, 476
Air of Schools, 183
Albany, Duke of, 478
Albuminuria and Life Assurance, 259
Alcohol: Is it a Food? 71
Alcoholism, Curiosities of, 119
Alnwick Infirmary (W.), 379
Ambulance, London, 451
Ambulance, Red Cross, 381, 444
American Doctors, The Making of, 234
Anæmia, Modern Aspects of Pernicious, 323, 389
Anæsthesia and Temperature, 431
Anarchists, Are A. Lunatics? 347
Animal Extracts, Treatment of Disease by, 345
Annus Medicus (1893), 209
Anxious Succession, An, 145
Appendicitis, 117, 148, 288
Appointments, see Vacancies
Around the Hospitals, 2, 18, 50, 98, 114, 146, 162, 178, 254, 276, 320, 342, 364, 386, 408, 430, 452, 474
Ars Longa, 193
Art, A Little More, 167
Art and Disease, 214, 256, 278, 302, 322, 366, 410, 432
Art and Disease (C.), 382
Artistic Deformity, 302
Aspects of Death, 410
Asylums in India, 44
Asylums, Some Scotch (W.), 225
Ayr County Hospital (A.H.), 320
Bacillus Prodigiosus in the Pantry, 215
Bacteria Underground, 38
Bedrest, 227
Bedsteads, 271
Beef, Butchers, and Buyers, 167
Belfast Hospital for Consumption (A.H.), 320
"Benefits Forgot," 451
Berkefeld Filter, 359
Biology, Modern, 37, 69, 100, 133
Biology, The Microscope in Relation to Modern, 3
Birds and Big Boys, 183
Birmingham General Hospital (C.), 79
Book World of Medicine and Science, The (books, magazines, and pamphlets, reviewed or noticed, and articles relating to)—
Acland and Ruskin. The Oxford Museum, 493
A Mere Don. Aspects of Modern Oxford, 405
Blades. How to Correct Printers' Proofs, 449
Boxall. Antiseptics in Midwifery, 295.
Bramwell. Atlas of Clinical Medicine, 493
Bütschli. Protoplasm and Microscopic Forms, 471
Campbell. Headache and Other Morbid Cephalic Sensations, 405
Chemistry, Meteorology, and Geology. Transactions of the Sanitary Institute, 229
Dane. Students' Dictionary of Medicine, 361
Endowment of Public Practitioners, 251, 317
February Reviews, 339
Flynn. Normal Labour, 493
Gasquet. The Great Pestilence, 427
Gynæcology, Student's Handbook of, 295
Hartridge. The Opthalmoscope, 427
Howden. Index Pathologicus, 449
January Reviews, 229
Kelly. The Medical Directory, 361
King. The Supernatural, 273
Kneipp. Thus Shalt Thou Live, 383
Maxwell. Life of W. H. Smith, 295
Meyer. The Child, 471
Paine. Pauperism in Great Cities, 361
Turner-Turner. Life in the Backwoods, 449
Vincent. Elements of Hypnotism, 383
Wade. On Gout, 229
Wall. Asiatic Cholera, 339
Wilson. Myxœdema, 273
Bradford Infirmary (A.H.), 386
Brain, Diseases of the, 195, 257
Brain Scavengers, 479
Bread, Germ, White, 262
Bridgwater Infirmary (A.H.), 162
Bristol General Hospital (W.), 423
Bristol Royal Infirmary (W.), 401
Bristowe, Dr., and His Friends, 7
Bristowe, Dr., Presentation to (W.) 15
Bucks General Infirmary (A.H.), 452
Cake, Almond Pound, 204
Cancer Researches, 23
Carbolic Disinfectants, 92
Caries of the Spine (C.), 12
Carriage of the Dead by Railways, 167
Case for Medical Discipline, 22
Cell Division, 133
Cells, Minute Structure of, 100
Cellular Structure of Living Organisms, 37, 69
Censorship, A New Surgical, 457
Central London Throat and Ear Hospital (A.H.), 18.
Certification (Death) and Murder, 49, 119
Charcot, J. M., 212
Chelsea and the Preventive Institute, 457
Chemical and Empirical Therapeutics, 35
Chester General Infirmary (A.H.), 452
Child of Crime, The, 4
Children, Other People's, 213
Chloral Hydrate Poisoning, Treatment of, 255
Chloroform Collapse, Treatment of, 475
Chloroform Commission, Net Gain of the, 361
Choler, a Dissemination of, by Flies, 38
Christmas Cheer, 180
Clark, Sir A., Illness of, 55
Clark, Sir A. (Memorial Notice), 81, 212
Clark, Sir A., A Monument, 253
Cochin Hospital, Paris (W.), 314
Complaints Against Country Hospitals, 199
Conference, International Sanitary, 325
Construction Notes (W.), 16, 31, 78, 144, 176, 207, 337, 359, 492
Consultants and General Practitioners, 65
Corpus Sanum, The, 344
Cottage Hospital Construction (W.), 96
Cremation at the Church Congress, 23
Criminal Children, Who Creates? 21
Cruelty in Irish Parents, 55, 79
Cupboards, Ward (W.), 293
Darwinism. Is Darwinism a Dying Faith? 363
Dead, Carriage of the, 167
Death Certification and Murder, 49, 119
Democracy and the Hospitals, 164
Derby Royal Infirmary (A.H.), 98
Derbyshire Hospital for Sick Children (A.H.), 114
Diabetes, Diet for, 199
Diabetes, Modern Aspects of, 5, 19
Diphtheria and Flats, 87
Diphtheria and Infective Endocarditis, 116
Diphtheria, Scarlet Fever, and Sore Throat, 300
Diphtheria, Therapeutics in, 99
Disease and Locality, 84
Disease and Sanitation, Incidence of, 68
Disease of Dietetic Origin, 169
Diseases of the Brain due to Diseases of the Ear, 195, 257
Disinfector, Steam (W.), 174
Diuretin and Diuresis, 343, 387
Dobbin, Mr. H. (W.), 63
Dr. A., Dr. B., Dr. C., 39
Doctor or the Chemist, The, 413
Doncaster Infirmary (A.H.), 114
Dover Hospital (A.H.), 342
Drink Cure at Dundee, 347
Drugs and their Uses, 411
Drugs, *See New Drugs, &c.*
Dumfries Infirmary (A.H.), 342
East London Hospital for Children (C.), 48
Ecclesiastical Evolution, 199
Edinburgh Royal Infirmary (W.), 125
Edinburgh Royal Infirmary (A.H.), 386
Editor's Letter Box, 12, 32, 48, 79, 96, 155, 172, 204, 272, 294, 316, 382, 400, 444
Educational Treatment of Idiots, 454
Empirical and Chemical Therapeutics, 35
Endocarditis and Diphtheria, 116
England and her Christmas, 177
Essex and Colchester Hospital (A.H.), 364
Evening Out-patients, 71, 369
Examination Paper (Edinburgh), 296
Famous Poisoners in Fiction, 4, 196
Field of Science, The, 6, 54, 70, 86, 102, 118, 134, 150, 166, 198, 236, 258, 298, 324, 368, 412, 434, 456
Alcohol as a Dressing, 6
Algeria and Rabies, 258
Ambulances in St. Louis, U.S.A., 166
American Post and Disease Germs, 6
American Precautions Against Cholera, 134
Army Medical Staff, Training of, 150
Bacillus Diphtheriæ and Milk, 258
Bacteria in Sea Water, 118
Bakeries, London, 434
Beer at Banstead Asylum, 258
Beri-beri, 6 (*and see p.* 488)
Briquettes, Dangers of, 368
Bullets and Infection, 102
Bureau of Health, U.S. National, 456
Burton, Sir R., and the Monkeys, 198
Canals as Sewers, 258
Cats and Infection, 6
Chelsea Scientific Institute, 456
Cholera Bacilli, Experiments with, 324
Cholera Bacilli and the Post, 298
Cholera, Precautions Against, in India, 118
Cholera, Precautions Against, by the U.S., 134
Cigarette Papers Made of Hospital Lint, 102
Claybury Laboratory and Museum, 236
Coccus (Microbe) in America, 324
Coffee and Tea, 412
Field of Science (*continued*)—
Convict Prisons, Good Sanitary State of, 118
Corpses, Theft of, 298
Cremation in Paris, 118
Crematorium at Cardiff, 456
Dairies in Victoria, Inspection of, 134
Democracy (Social) and the Leipzig Doctors, 456
Ears Made Out of a Man's Arm, 456
Electric Light Experiment, Remarkable, 412
Electro-Therapeutic (American) Association, 236
Epilepsy and Errors of Refraction, 412
Eucalyptus Plant, 86
Expulsion of Disease (A Superstition), 198
Eysight of Calcutta Children, 6
Female Beauty and Education, 368
Hæckel, Bust of, 324
Hermite's Experiments, 166
Hypnotism, its Uses, 102
Hypnotism in Russia, 102
Indiarubber, Supply of, 118
Inter-Hospital Communication, 86
Lead Pipes, 70
Leprosy and the Indian Commission, 54
Liquid Air, 134
Lungs (Human) Transformed into Pure Carbon, 236
Magnetic Healer at a Christmas Party, 368
Marseilles Anti-Rabic Institute, 258
Match Factories and Health, 324
Medical Congress, Free, 198
Medical Congress in India, Proposed, 198
Medical Congress, Norwegian, 102
Medical Fees in Russia, 150
Medical Officers of Health and Stipends, 7
Morgue, The, 70
Morphia, Misuse of, in France, 86
Morphine Ordinance of Hong Kong, 134
Muscle and Nerve Experiments, Yale, 258
Opium and Life Assurance, 368
Opium in Victoria, 150
Owen Memorial, 198
Oxygen, Effects of Breathing, 150
Pasteur Institute, 54
Pasteur Institute at Constantinople, 456
Phthisis, Precautions Against, 456
Physical Recreation, 6
Preventive Measures: An American Caution, 298
Pulmonary Disease and Ventilation, 6
Rabies, *see Algeria, Marseilles*
Reading in Vehicles, 102
Refuse, Disposal of City, 54
Russia and International Medical Congress of 1896, 298
Russia, Hypnotism in, 102
Russia, Superstition in, 258
Russia, First Vaccination in, 118
Russia, Weights and Measures in, 236
Russia, Women Doctors in, 324
Sanitation Sunday. 456
Schnebelin (Explosive), 324
Seismograph, New, 150
Sewers Free from Microbes, 412
Smoking Prophylactic, 102
Smoking and Human Development, 118
Spelling, American, 86
Suicide in France, 134
Tattoo Marks, Suggestion As To, 368
Tea and Coffee, 412
Tuberculosis and Fish, 412
Unremunerative Science, Note on, 134
Vaccination Long Known in India, 368
Vaccination, First, in Russia, 118
Ventilation and Pulmonary Disease, 6
Water, Worship of, 54
Worthing Sewerage, 166, 236
Yale Experiments on Nerve and Muscle, 258
Figures that Feel and Work, 181
Filter, Berkefeld, 359
Flies, Dissemination of Cholera by, 38
F ood Warmer (W.), 403
Free or Sober, 161
French Hospital (A. H.), 254
Gas Stoves (W.), 62
Gastric Juice, Acidity of (its diagnostic value), 476
Gladstone's Eyesight, Mr., 457
Glasgow Royal Infirmary (A. H.), 364
Glasgow Western Infirmary (A. H.), 178
Graves' Disease, Modern Treatment of, 433
Great Achievements under Suffering, 478
Guard (Victoria) for Window Cleaning, 468
Guthrie Society, Westminster Hospital, 230
Healing of the Sick, The, 366
Henry VIII. to the Rescue, 369
Herbal Simples, The History and Capabilities of, 52, 280
Holmes, Mr. T., on John Hunter, 1
Hospital Accounts, Uniform System (W.), 126
Hospital Administration (W.), 16, 29, 45, 77, 269, 313, 335

Hospital Construction (W.), 47, 94, 110, 125, 141, 158, 247, 314
Hospital Festivals and Meetings (W.), 111, 127, 143, 159, 315, 337, 359, 383, 403, 425, 448, 469, 491
Hospital Finance (W.), 13, 226, 291, 380, 467
Hospitals for Infectious Disease, 173
Hospitals in India (W.), 60, 66, 175, 205
Hospitals, Military and Naval (W.), 157
Hospital Saturday and Street Collections, 34
Hospital Saturday Fund (W.), 293
Hospitals, Within the (W.), 61, 93, 109, 357, 379, 401, 423, 445, 489
Hospital Worthy, A (W.), 63
Howard Association, 39
Hunter, John, Mr. T. Holmes on, 1
Huxley and Clericalism, 7
Hydrogen Peroxide, Injection of, 67
Hysteria or Possession, 214, 256
Ideals Unrealised, Medical, 385
Idiots, &c., Educational Treatment of, 454
Imperial Typhoid: the Unemployed, 275
Incidence of Disease and Sanitation, 68
India, Asylums in, 44
India, Hospitals in (W.), 60, 66, 175, 205
India, Vaccination in (C.), 400
Indian Oculists, 71
Infection of Phthisis, 203
Infection and the Post, 298
Infectious Disease Hospitals in London (W.), 173
Inflammation. Modern Aspects of, 165
Injection of Hydrogen Peroxide into the Peritoneal Cavity, 67
Inoculation and Insusceptibility, 132
Insane, A New Method of Treating the, 20
Insane, Story of the, from Year to Year, 62, 96, 126, 207, 292, 336, 358, 424
Insanity and its Kinships, 407
Insects and Growing Fruit, 44
Instrument Table (W.), 31
Joint Disease (Tuberculous), Operatic Treatment of, 235
Journalists. Should Journalists Marry? 413
Jowett, Professor, 7
Kent and Canterbury Hospital (A. H.), 430
Kettle, Pendulous (W.), 403
Kilmarnock Infirmary (A. H.), 276
King's College Hospital (W.), 93
Kirkheaton Sanitary Matters, 119
Lame and the Blind, The, 322
Law, Medicine and Humanity, 33
Leaderless Medicine, 429
Learned Profession, A, 97
Leeds General Infirmary (C.), 79
Leeds Women and Children's Hospital (A. H.), 474
Leopold, Duke of Albany, 478
Leprosy at Trinidad, Report on (W.), 205
Life Assurance Medical Officers' Association, 303
Lincoln County Hospital (A. H.), 276
Local Government Board, Medical Work of, 36, 53, 68, 84, 116, 132
Locality and Disease, 84
Lockers (W.), 293
Locker, Glass-topped, 112
London. Shall London Have a University? 325
London Hospital (W.), 110
Longevity, Aids to, 391
Lunacy Blue Book (W.), 31
Lunacy Commissioners for Scotland. 35th Report, 174
Lunatics? Are Anarchists, 347
Maclean Hospital, Waverley, Mass., 247
Magdalen Hospital, Streatham (C.), 96
Malthus Redivivus, 103
Man, Study of, 101, 149, 197, 301, 367
Manchester Royal Infirmary (A. H.), 474
Manchester Southern Hospital (A. H.), 98
Manure Conundrum, The, 53
Massage in Edinburgh, 391
Materialism, Madness, and Poor Humanity, 281
Mattei Remedies (C.), 155
Medical Confessional, 215
Medical Defence Union and the Protection Society (C.), 155, 172, 204
Medical Discipline, A Case for, 22
Medical Lovers' Quarrel, 151
Medical Officers' (Life Assurance) Association, 303
Medical Programme, 231
Medical Progress and Hospital Clinics:—
  Abdominal Surgery, 222
  Anthrax, 488
  Antiseptics, 331
  Antiseptics (Ophthalmology), 266
  Aural Suppuration, 25, 41, 57
  Beri-beri, 488 (*see p.* 6)
  Blepharitis, 223
  Bone, New Growths of, 219
  Bronchitis, Plastic or Fibrinous, 240
  Cervix Uteri, Cancer of, 372
  Children, Diseases of, 439
  Cholera, 486
  Chorea, 201
  Colotomy, Inguinal, 482
  Conjunctivitis, 266
  Corneal Ulcers, 267
  Dangers of the "Rectangular Position," 74
  Diarrhœa, Summer, of Children, 52
Medical Progress and Hospital Clinics (*con.*)—
  Dilated Stomach, 123
  Diphtheria, 202, 285, 373
  Drainage in Surgical Treatment, 262
  Drainage of Wounds and Cavities, 283
  Empyema, 58
  Empyema of the Antrum, 416, 437
  Epiphyses, Injuries to the, 354
  Erysipelas, 42, 107, 153
  Eyes, Inflamed, 327
  Fingers, Contraction of the, 138
  Flatfoot, 185
  Fractures, Simple and Compound, 328, 351
  Fractures and Dislocations, 332
  Genito-urinary Organs, Surgery of the, 397
  Hæmoptysis, 287
  Hæmorrhage (Post-partum), 484
  Hare-lip, 73
  Hernia, 121, 123, 377
  Hip-joint Disease, 170
  Hirsuties and Trichiasis, 349
  Insomnia, 306
  Intestines, Surgery of the, 308
  Iritis, 267
  Jaundice, Catarrhal, 438
  Joints, 354
  Laparotomy, 218
  Lavage of the Intestinal Tube, 171
  Leprosy, 488
  Liver and Gall Bladder, 263
  Lungs, Diseases of the, 329
  Maniacal Delirium, Acute, 137, 153
  Meningitis, Spasm in the Diagnosis of Acute, 217
  Menstruation, Painful, 394
  Myxœdema, 91
  Nævi, 10
  Necrosis of the Lower Jaw, 239
  Neuroma of the External Popliteal Nerve, 460
  Nerve Diseases, 395
  Nervous Diseases, 352
  Ophthalmia (Ophthalmology), 267
  Ophthalmology, 223, 266
  Optic Atrophy, 267
  Otorrhœa, 25, 41, 57
  Pannus, 267
  Periostitis, Acute, in Children, 305
  Peritonitis, Tubercular, 419
  Peritonitis, Tubercular, Complicated, 218
  Pharyngo-mycosis, 373
  Pleurisy, 220
  Pneumonia, 105, 307, 459
  Pott's Disease, Plaster Bed for, 261
  Preparation of Patients for Operation, 371
  Rectangular Position (Elbow-joint), Dangers of, 74
  Retina, Detached, 267
  Retinitis, 267
  Rheumatism, Acute, 461
  Rhinitis, 464
  Rhinology, 463
  Ringworm, 26, 42
  Salophen, 268
  Serum and Antitoxin Treatment of Tetanus, 268
  Skin Affections, 393
  Skin Grafting, 376
  Stomach, Surgery of the, 245
  Straight Position in Injuries of the Elbow-joint, 89
  Tetanus, 268, 486
  Throat, Diseases of the, 373
  Thyroid Feeding in Skin Diseases, 284
  Thyroid Preparations, 139
  Toenail, Ingrowing, 9
  Tonsils, Diseases of the, 398, 420
  Trichiasis, 349
  Typhoid, 485
  Tuberculosis, Pulmonary, 243, 264
  Uric Acid, Formation, Effects, 481
  Vermiform Appendix, Diseases of the, 288 (*and see pp.* 117, 148)
  Yellow Fever, 487
Medical Protection and Defence, 139
Medical Protection for School Morals, 473
Medical Protection, The London and Counties M.P. Society, 12
Medical Science, Practical Aspects of, 346, 390
Medical Work of the Local Government Board, 53, 68, 84, 116, 132
Medical Workers and Medical Thinkers, 237
Medicine Cupboard (W.), 142
Medico-psychology and Psychialtry, 477
Microbe or the Missionary, The, 303
Microscope in Relation to Modern Biology, The, 3
Middlesex Hospital (W.), 109
Middlesex Hospital Improvements (W.), 62
Middlesex Hospital Ward Fittings (W.), 337
Midwifery Case, The Compactum, 242
Midwives' Registration Association (C.) 272, 294
Midwives, Should, be Registered, 259
Military and Naval Hospitals (W.), 157
Mincepie Memoranda, 237
Minds of Nations, 367
Minute Structure of Cells, 100
Mixture, An Incompatible, 479
Moseley Hall Convalescent Home for Children (A.H.), 162
Munificent Gift, A. (C.), 294
Narcotics, Animal Temperature Under, 299
National Gallery, In the, 432
Navy, Health of the, 341
Newcastle-on-Tyne New Home for Incurables (W.), 47, 94
Newcastle Royal Infirmary (A.H.), 452
Newcastle Royal Infirmary (W.), 489
New Drugs, Preparations, and Appliances, &c.:
  Acetanilide, 421
  Agathin, 246
  Alumnol, 444
  Antipyrin, 421
  Benzosol Tabloids, 444
  Bismuth, New Preparation, 443
  Borax and Bromides in Epilepsy, 76, 92
  Borax Preparations, 92
  Bougie, Miller's, 92
  Caffeine, 465
  Calcium Chloride, 311
  Carbolic Disinfectants, 92
  Champagne, Coca, 488
  ,, Sans Sucre, 488
  Champetier de Ribe Bags, 312, 334
  Chirata, 422
  Chloralose, 290
  Chromatic Dial, 466
  Clinical Chart, Block and Case, 108
  Cocaine in Ophthalmic Work, 27
  Creasote, 11
  Dermatol, 356, 444
  Diuretin, 443
  Exalgin, 399
  Florador Food, 312
  Gallobromol, 443
  Gelatines, Unna's, 334
  Glenfield's Fluid, 92
  Harvest's Lentil Food, 155
  Hercules Horse-action Saddle, 400
  Hydrastinine, 311
  Hypnal Tabloids, 444
  Hypodermic Syringe, 356
  Inhaler, Silk's, 356
  Iodopyrin Tabloids, 444
  Isinglass and Gelatine (Swinborne's), 422
  Kingzett's Sulphur Candles, 333
  Lynch and Co.'s Catalogue, 378
  Malvern Water, 444
  Medicine Glass, New, 400
  Methylacetanilide, 399
  Microbene, 488
  Midwifery Case, The Compactum, 242
  Moyrapuama, 355
  Oatmeal, Inglis's, 124
  Ophthalmic "Tabloids," 466
  Ozonised Digestive Tea, 312
  Pads, "Leicester," 466
  Perchloride of Mercury, 399
  Phenacetin, 421
  Serum Therapy, 442
  Soaps, Antiseptic, 399
  Tobacco, Pure Turkish, 422
  Trional and Tetronal, 334
  Uric Acid Solvents, 108, 124
Northampton General Infirmary (A.H.), 50
Northampton, Hospital Week at (C.), 316
North Staffordshire Infirmary (A.H.), 178
Notes and News: 8, 24, 40, 56, 72, 88, 104, 120, 136, 152, 168, 184, 200, 216, 238, 260, 282, 304, 326, 348, 370, 392, 414, 436, 458, 480
Nottingham General Infirmary (A.H.), 146
Object Lessons from Lanarkshire, 55
Oculists, Indian, 71
Ophthalmic Medicine and Surgery, Modern Progress in, 83, 115, 147, 179, 233, 277, 321, 365, 409, 453
Opium and Life Assurance, 368
Opium Problem, The, 297, 382
Out-patients, Evening, 369
Out-patients, Evening, 71, 369
Oxygen, Inhalation of, for Therapeutic Purposes, 131, 163 (*and see p.* 150)
Oxygen, Modes of Administering, 211
Oxygen, On the Administration of, 163
Pain in Medicine, Modern Aspects of, 51, 85
Pain: Is Pain Necessary? 87
Paris, Cochin Hospital (W.), 314
Parkes, Dr. L., 457
Parkwood Convalescent Home (W.), 141, 158
Pendlebury Hospital for Sick Children (A.H.), 408
Perth City and County Infirmary (A.H.), 474
Phthisis, Infection of, 203
Pirated Inventions (C.), 48
Poisoner and Physician, 196
Poisoners, Famous in Fiction, 4, 196
Poisoning by Choral Hydrate, Treatment of, 255
Poor in London, The, 270
Poor Man's Church, A, 135
Poor and the Poor Law, The, 455
Poplar Hospital (W.), 61
Poplar Hospital, Appliances at (W.), 227, 271, 292
Port Sanitation, Progress in, 103
Possession, Modified Forms of, 278
Possession or Hysteria, 214, 256
Poultice Warmer and Inhaler (W.), 14
Practical Aspects of Medical Science, 346, 390
Practitioners (General) and Consultants, 65

Preventive Institute and Chelsea, 457
Preventive Institute, The, Another Complexion, 303
Preventive Medicine, Undignified, 281
Private, For Doctors only, 281
Public School Discipline, 135
Puerperal Diseases and Sanitary Deficiencies, 224
Quackery: Should Civilisation Suppress? 319
Quarterly Review on Hospitals, 113
Red Cross Ambulance, 381, 444
Remedies Secret and Doctors' Consciences, 87
Reverend Nottingham Surgeon, A, 347
"Rights" in Hospitals, 215
Rotunda Hospital, Dublin (A.H.), 178
Royal Alfred Institution (C.), 316
Royal Edinburgh Hospital for Sick Children (A.H.), 254
Royal Free Hospital (A.H.), 386
Royal Free Hospital (C.), 32
Royal Hospital for Incurables, 129, 194, 232
Royal Southern Hospital, Liverpool (A.H.), 408
Salford Royal Hospital (A.H.), 146
Salicylic Acid for Preserving, 156
Salisbury's Despondency, Lord, 151
Salt (Sir T.) Hospital (W.), 379
Sanitation, Incidence of Disease and, 68
Sanskrit Medicine, 38
Scarlet Fever, 300
Schoolboy Students, 135
School Morals, Medical, 473
Schools, Air of, 183
Scientific Temperance, 388
Scotch Asylums, Some (W.), 225
Scraps and Gleanings, 16, 32, 48, 64, 80, 96, 112, 128, 144, 160, 176, 208, 228, 250, 272, 294, 316, 338, 360, 426, 448, 492
Secret Remedies, 87
Selection and Race Progress, 413
Self-Drugging, Some Dangers of, 259
Sheffield General Infirmary (A.H.), 2
Shipwrecked Mariners' Society, Special Appeal, 382
Sociology, Studies in Applied, 213, 234, 344, 388, 455
Sore Throat, 300
Southampton Free Eye and Ear Hospital (A.H.), 50
Southport Infirmary (A.H.), 430
Spoilt Patients, 237
Steam Disinfector, 175
Stockport Infirmary (W.), 379
Street Collections and Hospital Saturday, 34
Study of Man, 101, 149, 197, 301, 367
Surgery, Simple Methods in, 23
Sweet Charity's Guide to Christmas Givers, 186
Tangier as a Health Resort, 479
Teeth, Our Failing, 369
Temperance, Scientific, 388
Temperature and Anæsthesia, 431
Temperature (Animal) under Narcotics, 299
Therapeutics, Chemical and Empirical, 35
Therapeutics in Diphtheria, 99
Threepence Threefarthings a Day, 183
Toilet Trays (W.), 359
Toothbrush Drill, 325
Trinidad Leprosy Report (W.), 205
Tripod (W.), 227
Two Notable Doctors, 212
Tyndall, Professor, Death of, 151
Ulster Hospital for Women and Children (A.H.), 430
Undignified Preventive Medicine, 281
Unemployed, The, 277
University, Shall London Have a, 325
Uniform Accounts for Hospitals, 126
Vacancies and Appointments, Medical, 230, 252, 274, 296, 318, 340, 362, 384, 406, 428, 450, 472, 494
Vaccination, 36
Vaccination in India (C.), 400
Vaccination, Who Invented (C.), 400
Ventilation and Ventilators, 315
Ventilation, Window, in Sick Rooms (W.), 206
Victoria Hospital, Swindon (A.H.), 18
Village Poor and London Hospitals, 176
Ward Fittings at Middlesex Hospital (W.), 337
Washstands (W.), 80
West Ham Hospital (W.), 357
West Ham Hospital (C.), 79
Whisper to Women, 39
Who Creates Criminal Children? 21
Window, A New, 446
Window-cleaning Guard, The Victoria (W.), 468
Within the Hospitals. *See Hospitals.*
Worthing Infirmary (A.H.), 2
Wrexham Infirmary (W.), 445

### THE SPECIAL NURSING SUPPLEMENT (MIRROR).

Accident, An Unfortunate, cxxv
Africa (South), Notes from, cclvi
After Care Association, clvii
American Letter, Our, lxvii
Annual Problem, An, cxxiii
Appointments, v, xviii, xxv, xxxviii, xlviii, lxxv, lxxxviii, cxv., cxlix, clxiii, clxxix, clxxxiv, ccviii, ccxix, ccxxx, cclx
Appointments, Minor, vii, xvii, xxvii, xxxv, lxxiii, xcvi, cv, cxlix, clv, clxiii, clxxxiii, cxcvi, ccvii, ccxvi, ccxxvii, ccxxxix, cclx
Australian Notes, ccxxxviii
Bangor, Trained Nurses at, ccvii
Belgrave Hospital for Children, lvi
Bird Show, A, xlvi
Book World for Women and Nurses, The, ix, xix, xxix, xxxix, xlix, lix, lxix, lxxix, lxxxix, xcix, cix, cxix, cxxix, cxl, cl, clx, clxx, clxxx, cxc
Canadian Notes, clv
Canaries, In the, lxxxviii
Causerie from New England, lviii, lxxvii, xcv, cvi, cxxviii, cxxxv
Cholera, Lecture on, ccvii
Christmas, An Annual Problem, cxxiii
Christmas at Biarritz, cxvi
Christmas Books, cxii
Christmas Carol, cxviii
Christmas Competitions, Our, cxxvii
Christmas Entertainments, cxxvi, cxxxviii, cxlvii
Christmas, How Bobbie Spent, cxviii
Christmas, Nellie's, cxvii
Christmas in India, cxxv
Christmas in Northern India, cxxv
Christmas in New York, cxxiv
Christmas in a Workhouse Infirmary, cxvi
Christmas with the Nuns, cxxviii
Christmas Day: The District Nurse, cxiv
Christmas Day in Japan, cxvii
Christmas Day with a Private Case, cxv
Competition, The Novel, xvii
Congress, International Medical, at Rome, ccliv
Consumption, The Prevention of, Amongst the Poor, clviii
Co-operation, The Nurses', clxxxiv
Cottage Hospitals in the West Country, lxxxvi
Death by Misadventure, clvii
Death in our Ranks, lxiii, cxlv, clxv
Difficulties of Private Nursing, ccxliv
Diphtheria, Lecture on, ccxxv
District, In the, xxv
District Nursing, cxxxiii, cxliv, cliv, clxiv, clxxiv, clxxxiv, cxcix, cciv
District Nursing, Thoughts on, xxxiv
Doctor, Trials of a, cviii
Everybody's Opinion, vi, xvi, xxvii, xlvii, lvii, lxvii, lxxvi, lxxxvii, xcvii, cvi, cxxviii, clxxxvii, cc, ccx, ccxx, ccxxx, ccxl, ccl, cclx
Examinations, Our, xxiv, lxxvii, cxxxiv, clxxvi, ccxxvii
Experiences of a Lecturer in a Rural District, xcviii
First Aid in Accident Cases—Programme of the Argentine Society, cxxxvi
Fund for Nurses, J. S. Morgan Benevolent, cclvii
Fund for Nurses, Royal National Pension, ccxlv
German Hospital, Dalston, clxviii
Glasgow Royal Infirmary, cxlv
Groat's Worth of Wit, A, xlviii, ccxxix
Guild of St. Luke, cviii
Guy's Hospital Trained Nurses' Institute, ccxlvii
Health Resorts for Consumptive Patients, ccxvii
Help the Nurses to Help the Sick, cxiii
Heredity, Nervous, in Villages, ccxlix
Heroines who nurse us, The, cl
Holiday Expeditions, xxviii
Hospitals, Irish, vi
Hospitals in Switzerland, xxxv
Infectious Hospital, Life in an, cv
Insanity, The Beginnings of, ccxiv
Irish Hospitals, vi
Italy, Sick Nurses in, clxxv
Lecture on Cholera, ccvii
Lecture on Diphtheria, ccxxv
Lectures to Ladies, xxxviii
Lectures at St. George's Hospital, ccv, ccxv, ccxliv, cclvi
Libraries for Nurses, xvii, xxxv
Male Nurses, The Training of, ccxxxv
Massage in Java, ccxxiv
Massage, Primitive, clxviii
Medical Women. Can Medical Women Nurse? ccxxv
Medico-Psychological Association of Great Britain and Ireland, lxxxvii, xcviii
Melbourne Letter, Our, ccxviii
Melbourne, Notes from, xciii, cclv
Mental Nursing, clxxiii
Midwifery Training at the Bristol Royal Infirmary, clxxix
Minor Appointments. *See Appointments, Minor*
Morgan (J. S.) Benevolent Fund for Nurses, cclvii
Muses' Looking-Glass, viii, xviii, lxviii, lxxviii, cxxxix, cxlix, clxix, clxxxix, cxcix, ccix, ccxix, ccxxix, ccxxxix, cclix
Nervous and Insane, On nursing the, cxliii, cliii, clxiii, clxxiii, clxxxiii, cxciii, cciii
Nervous System, On the Nursing of Diseases of the, iii, xiii, xxiii, xxxiii, xliii
New England. *See Causerie*
News from the Nursing World, i, xi, xxi, xxxi, xli, li, lxi, lxxi, lxxxi, xci, ci, cxi, cxxi, cxxxi, cxli, cli, clxi, clxxi, clxxxi, cxci, cci, ccxi, ccxxi, ccxxxi, ccxli, ccli
News from Toronto, ccvii
News from Victoria, xv
New York City Training School for Nurses, lxxxv
Nightingale Probationers, The, xxiv
Notes and Queries, vii, xviii, xxxviii, xlviii, lvii, lxv, lxxiv, cvii, cxxvii, clxviii, cxciii, ccix, ccxviii, ccxxvi, ccxxxv
Novelties and New Preparations, clxxxvii
Novelties for Nurses, xvii, xxxvii, xlviii, lvii, lxxviii, cvii, cxxxvi, cxlvi, clxviii, ccxvi, ccxxxvi, ccxlviii
Nurse, The Real and the Ideal, clxvii
Nurses, Domineering, cxcviii
Nurses, Old-time, cxcvii, ccvi
Nurses, Libraries for, xvii, xxxv
Nurses, A Quarantine Home for, xvii
Nurses, Training School for, lxxxv
Nurses' Confessional, The, cxcviii
Nursing Classes at Bow, clxxv
Nursing the Nervous and Insane, cxliii, cliii, clxiii, clxxiii, clxxxiii, cxciii, cciii
Nursing, On General, ccxiii, ccxxiii, ccxxxiii, ccxliii, ccliii
Nursing, Mental, clxxiii
Nursing, Private, Difficulties of, ccxliv
Nursing in Africa, ccxxxiv
Nursing in Argentina, civ, clxvi
Nursing in Ceylon, v
Nursing in India, clxxxiii
Nursing in Japan, xlv, lxxxvi
Nursing in Kimberley, xxxvii
Nursing in Labrador, lv, ciii
Nursing in Newfoundland, xlv, lv
Nursing in New York, ccxxv
Nursing in Paris, iv, xv, clvi
Nursing in South Africa, v, xxvi, xxxiv, cvii, ccxxxiv
Nursing in South Sea Islands, xcvi
Nursing in Switzerland, lvi, lxv, lxxxv, xciv
Nursing in the United States, xiv, xliv, liv, lxiv, lxxv, lxxxiv, cxlvi, clxxvii, clxxxvi, ccxxxvii
Nursing World, The. *See News.*
Observation, A Few Remarks on, cxxxiv
Ovariotomy Patients, The Nursing of, clxxvi, clxxxv
Patey, Madame, ccxxvi
Pension Fund for Nurses, Royal National, ccxlv
Presentations, lxvi, cvii, cxxv, cxxxiv, clxxxvi, ccxxvii, ccxlix
Prince Alfred's Hospital, Sydney, lxvi
Prince Alfred's Hospital, Sydney, Nurses' Home, cxcv
Queen Victoria Institute for Nurses, cxl
Read, What a Nurse Should, ccxlviii
Reading to the Sick, For, vii, xxvii, xxxvii, xlvii, lvii, cvii, cxxvii, cc, ccviii, ccxviii, ccxxviii, ccxxxvii, ccl, cclx
Royal British Nurses' Association, xliii, cv, cclix
Royal National Pension Fund for Nurses, ccxlv
Small-pox Case, An Isolated, iv, xvi
St. George's Hospital, Lectures at, ccv, ccxv, ccxliv, cclvi
St. Katharine's Hospital, cxxix
St. Luke, Guild of, cviii
St. Michael's Home of Rest, Lyme Regis, clxxv
St. Thomas's Hospital Chapel, ccliv
Stomach Diseases, On the Nursing of, liii, lxiii, lxxiii, lxxxiii, xciii, ciii, cxiii
Stray Visitors, clxv
Stroll Round the Wards, A, cxxxvii
Sunderland Infirmary, clxxv
Sussex County Hospital, cxxxviii
Teachers, Modern (Mrs. Sarah Grand), clxxix
Toronto, News from, ccvii
Trephining in the Stone Age, Professor Horsley on, ccviii
United States of America, The, xxxvi
Wants and Workers, viii, xvii, xxviii, xlvii, lxvi, lxxiii, lxxxiii, xcvi, ciii, clxxxiii, cxcvi, ccvi, ccxl, ccl
West Country, Cottage Hospitals in the, lxxxvi
Where to Go, xvii, xlvii, liii, lxviii, lxxvii, cvii, cxlviii, clxv, clxxviii, cxcix, ccxix, ccxxx, ccxlix
Women's Volunteer Medical Staff Corps, ccix, ccxxxvi
Women's Work, xlvi, lxiv, lxxiv, lxxxiv, xciv, clvi, clxvii, clxxviii, clxxxviii, cxcvi, ccxvi, ccxxvi, ccxxxvi, cclv
Worthing, Nurses at, lvii
Worthing, Queen's Nurses at, ccxlix

Printed by W. H. and L. Collingridge, "City Press," 148 and 149, Aldersgate Street, London, E.C.

**OUR NEW OFFICES,**
428, Strand, W.C., will henceforth be the Office of this Journal.

**Notices of Births, Deaths, and Marriages** are inserted in this column at a charge of 2s. 6d. for an announcement not exceeding four lines.

### NOTICE TO CORRESPONDENTS.

All MS., Letters, Books for Review, and other matters intended for the Editor should be addressed The Editor, The Lodge, Porchester Square, London, W.

The Editor cannot undertake to return rejected MS., even when accompanied by stamped directed envelope.

**Notice to Advertisers and Subscribers.**

All Advertisements, Orders for Copies of The Hospital, and Business Communications should be addressed to The Publisher, *not to the Editor*, The Hospital, 428, Strand, W.C.

The Cost of Subscription, post free, is as follows:—Per week, 2½d.; per quarter, 2s. 6d.; per half-year, 5s.; per annum, 8s. 6d. Foreign:—Per Annum, 10s. 8d.; payable, in all cases, in advance.

**"Burdett's Hospital Annual," 1893.**

Fourth year of publication. 700 pp. Boards, gilt lettering. A most complete guide to British, Colonial, Indian, and American Hospitals, Dispensaries, Nursing and Convalescent Institutions, and Asylums. Price 5s. Published by The Scientific Press (Limited), 428, Strand, W.C.

**NOTICE.—The Index for Vol. XIII. (ending March, 1893) is on sale at the Office of this Journal, price 2½d., post free.**

### Notes and News.

### HOSPITAL ADMINISTRATION.

### CONSTRUCTION NOTES.

### SCRAPS AND GLEANINGS.

The number of accident cases treated at the Scarborough Hospital since March 1st has been upwards of 1,000.

Hospital Saturday at Hastings and St. Leonards did not produce such satisfactory results as the collections of former years.

The Queen has graciously sent 137 bottles of wine to the Great Northern Central Hospital, Holloway Road, N., for the use of the patients.

The annual collections on behalf of the Newbury District Hospital and Dispensary took place recently; the total result achieved was £100, being a slight decrease on last year's collection.

For the purpose of raising the money still required for the building of the Derbyshire Royal Infirmary, parochial meetings are being held to organise house to house collections in each of the parishes.

A satisfactory report has lately been issued by the governors of the West Bromwich Hospital, showing a balance in hand on the year's expenses; a debt, however, still remains on the new building fund.

Rostellan Castle, Aghada, the residence of Lady Hennersy, was recently the scene of some interesting amateur theatricals, whose object was pecuniary assistance to the Eye, Throat, and Ear Hospital at Cork.

It has been decided by the Committee of the West of Scotland Convalescent Seaside Homes at Dunoon to add a new wing for women. When this addition is completed there will be a provision of 250 beds in the establishment.

A special meeting of the Westbury-on-Severn Local Board was recently called to consider the Infectious Diseases Hospital question, and to affect some agreement with a joint board for the general maintenance of the establishment.

At the expiration of September Leith Hospital ceased to be used for the treatment of fever patients, and it is proposed to utilise the space thus relieved as an extension of the hospital for the reception of general, medical, and surgical cases.

The Friendly and Trade Societies of Swindon recently held their annual demonstration in aid of the Swindon Victoria Hospital. After a procession through the town, a varied entertainment was given in the Great Western Railway Park.

The monthly paper of the Birmingham Hospital Saturday Fund states that at the last meeting of the Executive Committee resolutions were unanimously passed authorising immediate steps to be taken with a view to the establishment of a convalescent home for women.

An appeal on the part of Mr. Burroughs, the originator of the scheme for erecting a cottage hospital at Dartford, has as yet met with little response. Mr. Burroughs, had promised to give £1,000 if that sum met with a corresponding subscription of £2,000 from other sources.

An urgent appeal is being made for subscriptions to the Brighton and Sussex Throat and Ear Hospital Building Fund. The plans for the institution are ready and the site secured, but building operations cannot be commenced until more contributions can be secured.

The total amount of bequests benefiting the Belfast Royal Infirmary during the last quarter has been £570. The infirmary maintains its usual efficient condition, but the expenditure is still in excess of the receipts; subscriptions are being urgently solicited to put this on a fresh footing.

The annual hospital parade of the Bath Friendly Societies took place on a recent Sunday. The amount collected in the streets was £22, which, with the collection at the special service, will be divided between the Royal United Hospital, the Mineral Water Hospital, and the Ear Hospital.

Last year's balance-sheet of the Committee of the Gloucester Dispensary shows a deficit of £30, while members' payments amounted to £500. Arrangements have been entered into whereby the District Nursing Society are allowed to attend patients when they have entered the dispensary.

From the recently issued balance-sheet of the Twickenham Friendly Societies' Hospital demonstration, it appears that after all expenses were paid £90 was realised by the late special efforts, which sum was divided between St. John's Hospital, Twickenham, and the Royal Hospital, Richmond.

A children's ward is to be added to the Grimsby Hospital to commemorate the Royal wedding, which will be called the Princess May's Children's Ward. The people of Grimsby have exhibited great generosity in subscribing to the institution, the Working Men's Committee alone having collected over £200.

Two dogs have recently been acting as collectors for charitable objects, the one being at Folkestone, where it obtained over £3 for the Victoria Hospital funds, the other trusty animal collected money at Brighton towards the scheme for alleviating the distress at Worthing caused by the typhoid fever epidemic.

It is proposed to hold an exhibition in London next summer, the net profits of which will be distributed among the London hospitals. The exhibition will include four sections, professional loan, trade, naval, and military, and is to consist exclusively of articles dealing with the history and practice of medicine and nursing in all its branches.

The annual report of Sea View House, the first home established twelve months since by the Women's Convalescent Home Association, has just been issued. Many distinguished names appear among the recent contributors to the Association, which well deserves the support of all interested in the working women and girls of England.

Bristol Infirmary has at the present moment a debt of £5,000, which the committee are appealing for subscriptions to lessen. The normal annual income is only £10,000, and the institution cannot be carried on under £12,000. The infirmary is one of the oldest medical charities in England, and has earned a claim to a greater support than seems to be forthcoming.

The number of cases treated at the Basingstoke Cottage Hospital, last year, reached a total of 69, of these 36 were discharged cured, 23 improved and relieved, 7 died, while 3 remained under treatment at the close of the year. The committee, in presenting their report, offer cordial thanks to the medical staff and to the matron for their valuable services.

### The Scientific Press List.

**HOSPITALS AND ASYLUMS OF THE WORLD.** By Henry C. Burdett, in four volumes and a portfolio of plans, £8 8s.

**A PRACTICAL HANDBOOK OF MIDWIFERY.** By Francis W. N. Haultain, M.D. 18mo. Roan, Gilt Top. About 250 pages. (Ready October 16th.) 5s.

**HOSPITAL SISTERS AND THEIR DUTIES.** By Eva C. E. Lückes. 2s. 6d.

**OPHTHALMIC NURSING.** By Sydney Stephenson, M.B., F.R.C.S.E. Crown 8vo. About 200 pages. Illustrated. (Ready October 16th.)

**BURDETT'S HOSPITAL ANNUAL AND YEAR BOOK** OF PHILANTHROPY, 1893. By Henry C. Burdett. 5s.

**THE THEORY AND PRACTICE OF NURSING.** By Percy G. Lewis, M.D. 4th Edition. 3s. 6d.

**ACCOUNTS, AUDIT AND TENDERS, FOR HOSPITALS** AND INSTITUTIONS. The Uniform System of. By Henry C. Burdett. 6s.

**MENTAL NURSING.** By William Harding, M.B. 2s. 6d.

**OUTLINES OF INSANITY.** By Francis H. Walmsley, M.D. Cloth 3s. 6d. Popular Edition 1s. 6d.

**HOW TO BECOME A NURSE, AND HOW TO SUCCEED.** By Honnor Morten. 4th thousand. Profusely illustrated. 2s. 6d.

**ART OF FEEDING THE INVALID.** By a Medical Practitioner and a Lady Professor of Cookery. 3s. 6d.

**ART OF MASSAGE.** By A. Creighton Hale. 70 illustrations. 6s.

**SURGICAL WARD-WORK AND NURSING.** By Alexander Miles, M.B. (Edin.), C.M., F.R.C.S.E. (In the Press.) 3s. 6d. net.

**MINISTERING WOMEN: THE STORY OF THE** ROYAL NATIONAL PENSION FUND FOR NURSES. By George W. Potter, M.D. 2s. 6d

**THE NURSE'S DICTIONARY OF MEDICAL TERMS** AND NURSING TREATMENT. By Honnor Morten. 5th thousand. 2s.

**COTTAGE HOSPITALS, GENERAL FEVER AND CON-**VALESCENT: THEIR PROGRESS, MANAGEMENT, AND WORK. By Henry C. Burdett. (Third Edition in the Press.)

**LONDON: THE SCIENTIFIC PRESS, LIMITED.—428, STRAND, W.C.**

### The Book World for Women and Nurses.

### PUBLISHERS' ANNOUNCEMENTS.

Price 2/6 Demy 8vo. 200pp. Profusely Illustrated with Copyright Portraits and Drawings. Price 2/6 Post Free. Post Free.

HOW TO BECOME A NURSE; AND HOW TO SUCCEED.

**A complete guide to the Nursing Profession for those who wish to become Nurses, and a useful Book of Reference for Nurses who have completed their training, and are looking for Employment in the United Kingdom and Abroad.**

Compiled by HONNOR MORTEN.

(*Author of* "Sketches of Hospital Life," "The Nurse's Dictionary," etc.).

CONTAINS Alphabetical List of the Chief Institutions in the United Kingdom and Abroad where Nurses are Trained and Employed; Fees, Qualifications and all Particulars; Information as to Application, Probation, Certification, Midwifery, etc.; Private, Infirmary, District, Asylum, Army and Navy, and Nursing Abroad; Male Nursing; Massage; Case-taking; Uniforms (Illustrated); Matrons; Nursing Societies and Guilds; Equipment; Examination Questions; etc., etc.; LIVES OF EMINENT NURSES (with Copyright Portrait of each):—Miss Florence Nightingale, Sister Dora, Miss Fisher, Mrs. Dacre Craven, Miss Lückes, and others.

Price HALF-A-CROWN (post free).

Publishers: The Scientific Press (Limited), 428, Strand, London, W.C.

Price 6d., Post Free. Demy 16mo. 2nd Edition. 7th Thousand. Price 6d., Post Free.

THE NURSES' CASE BOOK.

**For Use in Hospital as well as Private Nursing.**

*Suitable Size for the Apron Pocket.*

This Book is intended for the use of Nurses, that they may keep a brief record of their cases for their own instruction, as well as the record they have to keep for the medical attendant. Each sheet is ruled to last a week, and two sheets should usually be allowed for a case, though in typhoid it may be necessary to leave three, and in cases of common fracture one may be enough. It contains TWENTY-SIX COMPLETE TABLES, each being ruled to last ONE WEEK, for keeping an exact record of a Patient's condition, including Notes on TEMPERATURE, PULSE, RESPIRATION, MOTIONS, AGE, SEX, NAME, ADDRESS, OCCUPATION, TREATMENT, HISTORY, NAME OF MEDICAL ATTENDANT, NUMBER OF CASE, and DATE OF ADMISSION, &c., &c.

Nurses should combine together and order parcels of over a dozen to profit by the special discount terms—*i.e.*, 4s. 6d. PER DOZEN (POST FREE).

London: The Scientific Press (Limited), 428, Strand, W.C.

Now ready, crown 8vo., 5th Edition. Price 4s. net.

MANUAL OF OBSTETRIC AND GYNÆCOLOGICAL NURSING.

By FLEETWOOD CHURCHILL, M.D. Revised and Edited by THOMAS MORE-MADDEN, M.D., F.R.C.S.Ed. Obstetric Physician to Mater Misericordiæ Hospital; Master of the National Lying-in Hospital, Dublin.

"We can confidently recommend this revised work to all interested in the subject of which it treats."—*The Hospital.*

"Dr. More-Madden's Handbook . . . may be safely recommended as a reliable Text-book."—*Provincial Medical Journal.*

"Our opinion is that not only is it an excellent and complete work for the nurse's use, but many students and young practitioners would gain much that would be of use from its perusal."—*Medical Times and Hospital Gazette.*

Fannin and Co., 41, Grafton Street, Dublin.

NOW READY.

Catalogue of Books,

On nearly every description of Nursing, sent post free on receipt of postcard by The Scientific Press (Limited), 428, Strand, W.C.

Fourth Thousand, Cloth, Price 1s. 6d. nett, post free.

London: The Scientific Press (Limited), 428, Strand, W.C.

### BOOKS RECEIVED.

Fannin and Co., Dublin.

"A Handbook of Obstetric and Gynæcological Nursing." By the late Fleetwood Churchill, M.D., revised and enlarged by Thomas M. Madden, M.D.

Charles Griffin and Co.

"An Elementary Text-book of Biology." By J. R. Ainsworth Davis, B.A. (2 vols.)

"The Diseases of Childhood." By H. Bryan Donkin, M.D., F.R.C.P.

### PUBLISHERS' ANNOUNCEMENTS.

THE SCIENTIFIC PRESS

LIST OF NEW PUBLICATIONS

(READY OCTOBER 16th).

SURGICAL WARD WORK AND NURSING.
Demy 8vo, Cloth Boards, copiously illustrated. A practical Manual of Clinical Instruction for Students and Nurses in the Wards. Concisely, though comprehensively treated. By ALEXANDER MILES, M.D., C.M., F.R.C.S.E. Price 3/6 net.

HELPS IN SICKNESS AND IN HEALTH.
Crown 8vo., 2nd Edition, about 300 pages. By HENRY C. BURDETT.

A PRACTICAL HANDBOOK OF MIDWIFERY.
18mo, profusely illustrated with Original Cuts, Tables, &c., about 200 pp. By FRANCIS W. N. HAULTAIN, M.D. Roan, gilt top. Price 5/-

OPHTHALMIC NURSING.
Crown 8vo, illustrated. By SYDNEY STEPHENSON, M.B., F.R.C.S.E.

THE MOTHER'S HELP AND GUIDE TO THE DOMESTIC MANAGEMENT OF CHILDREN. By P. MURRAY BRAIDWOOD, M.D.

*FULL CATALOGUE POST FREE ON APPLICATION.*

LONDON: THE SCIENTIFIC PRESS, Limited, 428, STRAND, W.C.

Pocket size, 2s. post free, with numerous Illustrations, reduced from the larger work by the same Author.

A PRIMER OF THE ART OF MASSAGE (FOR LEARNERS).
By THOMAS STRETCH DOWSE, M.D., F.R.C.P.Edin.

"An excellent little book, giving the more important facts about massage. Will be useful."—*Dub. Med. Jour.*

Bristol: J. Wright and Co.
London: Simpkin and Co. (Limited).

Post free. 2/6 In handsome cloth boards, 150 pp., Illustrated. Post free. 2/6

Minstering Women:

The Story of the Royal National Pension Fund for Nurses.

*By GEORGE W. POTTER, M.D.*

"Is full of interest, and the sale of the book will be stimulated by the fact that the profits of the author are to be devoted to the 'Junius S. Morgan Benevolent Fund.' An excellent portrait of the Princess of Wales forms a suitable frontispiece."—*Liverpool Courier.*

"It should commend itself to all—women especially—who can appreciate the noble work undertaken by those true sisters of mercy and self-denial, our hospital nurses. The record is full of touching interest. . . . The book is well worth the half crown charged. . . . Every nurse should possess herself of a copy."—*The Lady.*

London: The Scientific Press (Limited), 428, Strand, C.W.

### Medical Vacancies and Appointments.

### APPOINTMENTS.

W. R. Berridge, M.R.C.S., L.R.C.P.Lond., appointed Medical Superintendent of the Infectious Diseases Hospital of the Blaby Rural Sanitary Authority. F. S. Boreham, L.R.C.P., L.R.C.S. Edin., appointed Medical Officer for the No. 5 District of the Market Harborough Union. J. Cowper, M.B., C.M.Edin., appointed Medical Officer of Health to the Shanklin Local Board. J. Craig, M.B., C.M.Edin., M.R.C.S.Eng., reappointed Medical Officer and Public Vaccinator for the Biddulph District of the Congleton Union. A. R. Douglas, L.R.C.P., L.R.C.S. Edin., M.P.C., appointed Assistant Resident Medical Officer at the Royal Albert Asylum, Lancaster. H. A. Eccles, M.B.Lond., M.R.C.S., appointed Assistant Resident Medical Officer at the London Fever Hospital, Liverpool Road, N. E. R. Evans, L.R.C.P.Lond., M.R.C.S.Eng., appointed Medical Officer of Health for the Newcastle-in-Emlyn Union. J. G. Gibbon, M.B., appointed Medical Officer for the Twelfth District of the Wisbech Union. J. E. Gould, M.D., L.R.C.P.Lond., M.R.C.S. Eng., appointed Medical Officer of Health for the Borough of Chesterfield. W. Hoffmeister, M.D.Heidelb., L.R.C.P.Lond., M.R.C.S.Eng., reappointed Medical Officer of Health to the Cowes Port Sanitary Authority. J. A. Hogg, M.R.C.S.Eng., L.R.C.P., appointed Resident Medical Officer to the Birmingham Workhouse Infirmary. C. Hunter, L.R.C.P., F.R.C.S.Edin., appointed Medical Officer for the No. 2 Dispensary, Carrickmore. J. W. Jackson, L.R.C.P.Edin., M.R.C.S.Eng., appointed Medical Officer for the Winchmore Hill District of the Edmonton Union. C. M. Kempe, M.R.C.S., L.S.A., reappointed Medical Officer of Health for the Shoreham Urban Sanitary District and Port. W. J. Leitch, L.R.C.P., L.R.C.S.Edin., appointed Medical Officer to the Clogher Workhouse. A. C. Lyon, M.D., appointed Medical Officer for the Waterbeach District of the Chesterton Union. W. O. Majoris, L.R.C.P., L.R.C.S.Edin., appointed Medical Officer for the Shelford District of the Chesterton Union. G. H. Moorhead, appointed Medical Officer for the No. 15 District of the North Bierley Union. G. R. Murray, B.A., M.B.Cantab., M.R.C.P.Lond., appointed Professor of Comparative Pathology in the University of Durham. M. J. Nolan, L.R.C.P., L.R.C.S.I., appointed Resident Medical Superintendent to the Downpatrick Asylum. W. P. Peake, L.R.C.P.Lond., M.R.C.S.Eng., reappointed Medical Officer for the First District of the Leicester Union. R. Prichard, M.D.Glas., appointed Inspector of Nuisances to the Glamorgan Rural Sanitary Authority. C. F. Pridham, B.A.Cantab, M.R.C.S.Eng., L.R.C.P.Lond., appointed Medical Officer for the Tenth and Eleventh Districts of the Wycombe Union. H. H. Pridie, M.B., C.M.Edin., appointed Medical Officer for the Wandsford District of the Stamford Union. J. Priestley, B.A., M.B. (R.U.I.), D.P.H. (R.C.S.Eng. and P.Lond.), appointed Assistant Medical Officer of Health for Paddington, W. J. Stewart, M.B., C.M.Glas., appointed Medical Officer for the Soothill Upper District of the Dewsbury Union. H. A. Stonham, M.R.C.S., L.R.C.P., appointed Medical Officer to the Ratcliff Workhouse. T. W. Thomas, M.R.C.S.Eng., L.S.A., appointed Medical Officer for the Caerphilly Urban Sanitary District of the Pontypridd Union. C. Tweedy, M.R.C.S.Eng., appointed Medical Officer of Health for Northallerton. D. Watson, M.B., C.M.Glasg., appointed Medical Officer for the Eleventh District of the Wisbech Union. R. H. Watson, M.A., M.B., C.M., appointed Assistant Physician to the Inverness District Asylum. J. B. Webb, M.R.C.S.Eng., L.R.C.P.Lond., appointed Surgeon to the Bristol Dispensary. J. Yuill, M.B., C.M.Glasg., appointed Medical Officer to the Local Lodge of Oddfellows at Kincardine.

### VACANCIES.

The following vacancies are announced this week, the amount of salary in each case being given after the name of the institution:

### Resident Medical Officers.

Fulham Union. Assistant Medical Superintendent, unmarried, doubly qualified. Salary £100 per annum, increasing £10 yearly to £130, with board, furnished apartments, attendance, and washing. Applications, on forms to be obtained of the Clerk, to T. Aplin Marsh, Clerk to the Guardians, Union Offices, Fulham Palace Road, Hammersmith, W., by October 14th.

Portumna Union, No. 2 Dispensary District. Medical Officer. Salary £40 per annum, with £5 yearly as Medical Officer of Health, together with registration and vaccination fees. Applications to Mr. L. Taylor, Honorary Secretary. Election on October 7th.

Rotherham Hospital and Dispensary. Assistant House Surgeon. Rooms, commons, and washing provided. Appointment for six months. Applications to the House Surgeon by October 19th.

**Non-Resident Medical Officer.**

Salisbury Infirmary. Dispenser. Salary £105 per annum, out of doors. Applications to the Secretary by October 13th.

### SALOL AS A MATERIAL FOR COATING PILLS.

The difficulty of securing a satisfactory coating of pills with keratin has induced Dr. G. Oeder to make trial of various other substances in its stead, and he has found that salol is well suited for the purpose. The object in view is to provide for the pills passing through the stomach without alteration and being acted upon only when they reach the intestine. Salol has already been recommended as a pill coating for this purpose by Ceppi and Yvon, but they proposed using it in the form of an ether solution. That mode of applicatian was not found to give good results, the deposit of salol upon the pills being too friable and readilyrubbed off. Dr. Oeder prefers to apply salol in a melted condition for coating pills, and the operation is carried out in an enamelled sheet iron tray, upon the bottom of which some powdered salol is melted over a spirit lamp or gas flame. The pills are then placed in the tray and rolled in the melted salol, sufficient heat being applied meanwhile to prevent solidification until the surfaces of the pills are coated with a thin layer. The heating is then discontinued and the rolling of the pills kept up for about one minute until they have sufficiently cooled. For thirty pills of average size the quantity of salol requisite is from a gramme to a gramme and a half, but if the pills are not sufficiently coated in one operation the treatment must be repeated. The pills should have a uniform translucent coating, free from cracks or bare places, and the quantity of salol on each pill need not exceed two centigrammes. Dr. Oeder states that he has succeeded in obtaining a sufficient coating with as little as five milligrammes, and even in the case of the largest sized pills the salol coating need not exceed one decigramme. In carrying out the operation the chief point to be observed is to avoid heating too much, as that would have the effect of decomposing the salol. The low melting point of salol (40°—43° C.) facilitates the operation, and if that temperature is not exceeded the substance may be repeatedly melted without undergoing alteration.—*Pharmaceutische Zeitung*, xxxviii., 527.

### PASS LISTS.

**Examining Board in England by the Royal Colleges of Physicians and Surgeons.**

The following gentlemen passed the first examination of the Board, under the "Five Years' Regulations," in the subjects indicated :—

Passed in Elementary Anatomy.—J. A. Archer, Mason College, Birmingham; H. J. M. D. S. Barker, Guy's; F. Barton, University College, Liverpool; A. St. J. Bateman, King's College; G. H. Bedford, Guy's; R. Brookes, Westminster; T. W. Byrne, University College, Liverpool; C. S. Cato, Westminster; M. B. Dawson, Middlesex; H. O. P. de Mirimonde, Yorkshire College, Leeds; J. C. Douglas, Middlesex; A. L. Edwards, St. Mary's; D. R. Edwards, University College, Liverpool; A. T. Falwasser, Guy's; T. B. Fawley, Firth College, Sheffield; H. W. Fox, Guy's; W. D. French, University College; C. Gasgoigne, St. Mary's; J. G. B. Gowlland, St. Bartholomew's; F. H. Hand, St. George's; W. H. Harland, Middlesex; G. W. Harrison, St. Thomas's; S. Harrison, Guy's; W. T. Hillier, Middlesex; W. Hodges, St. Thomss's; H. C. Jones, St. Mary's; E. H. Kitchin, Yorkshire College, Leeds; M. R. Mayer, University College, Liverpool; W. S. Maugham, St. Thomas's; A. Mercer, University College; S. L. C. Mondy, University College; C. C. Morgan, St. Bartholomew's; A. H. Norris, Owen's College, Manchester; J. Oldfield, St. Bartholomew's; G. O'Neill, Charing Cross; G. C. Owsley, Guy's; A. H. Parker, Charing Cross; H. J. Phillips, St. Thomas's; N. J. Roche, Charing Cross; H. A. Scholberg, St. Bartholomew's; A. F. Seacome, University College, Liverpool; H. Underhill, Mason College, Birmingham; J. H. Watson, University College, Liverpool; M. T. Whitehouse, Mason College, Birmingham; W. H. G. Whittuck, University College, Bristol; J. A. Wilson, King's College.

### THE EDINBURGH TRIPLE QUALIFICATION.

**Papers Recently Set.**

FIRST EXAMINATION.

Questions in Chemistry (three of which are to be answered).

1. What changes do the following substances undergo when heated: (*a*) Potassium chlorate, (*b*) ferrous sulphate, (*c*) potassium ferrocyanide, (*d*) oxalic acid, (*e*) calcium carbonate ?

2. How are the following substances prepared ? Give equations : (*a*) Phosphoric acid, (*b*) ammonia, (*c*) glycerine, (*d*) tartaric acid ?

3. Explain what is meant by fractional and by destructive distillation. Give an example of each.

4. How much oxygen is required completely to burn half a cubic foot of olefiant gas ($C_2H_4$) ? What would be the volume of carbonic acid formed at 17° C. and 700 mm. pressure ?

